# Prevalence of anaemia and associated factors among geriatric population from rural Mangalore: a cross-sectional study

**DOI:** 10.1186/s12877-025-06605-1

**Published:** 2025-11-19

**Authors:** Babburi SaiHarika, Rekha T, Ramesh Holla, Darshan BB, Nithin Kumar, Prasanna Mithra P, Bhaskaran Unnikrishnan, Aishwarya Narasimhan, Himani Kotian

**Affiliations:** 1 Department of Community Medicine, Kasturba Medical College Mangalore, Manipal Academy of Higher Education, Manipal, Karnataka India; 2 Department of Community Medicine, Chamarajanagar Institute of Medical Sciences, Chamarajanagar, Karnataka India

**Keywords:** Anaemia, Geriatric, Morphological features, Rural population

## Abstract

**Background:**

Anaemia is a major public health problem in all age groups. Anaemia in elderly individuals may result in several adverse health outcomes, including functional dependence and increased risk of therapeutic complications, falls, dementia, and death. Anaemia is often overlooked as symptoms such as weakness, fatigue, shortness of breath etc., are also associated with ageing. Hence, early detection of anaemia in elderly individuals is necessary to prevent delays in diagnosing potentially treatable conditions.

**Methods:**

This community-based cross-sectional study aimed to estimate haemoglobin level among the geriatric population, classify anaemia on basis of morphological features and assess the factors associated with anaemia. The study included elderly individuals above 60 years of age, with a sample size of 306 participants residing in rural Mangalore. Convenience sampling (non-random) was done from all the villages. After the Institutional Ethics Committee (IEC) approval, data was collected using a semi-structured, validated questionnaire. Blood was taken by venepuncture, later collected into EDTA tubes and further processing of samples was done in a laboratory. Descriptive statistics like median, interquartile range and proportions were used and association of anaemia was analysed by using Chi-square test.

**Results:**

The mean age of the study participants was 67.2 ± 7.1 years. The median (IQR) haemoglobin level was 14.0 (12.9–15.1) g/dL in men and 12.8 (11.9–13.5) g/dL in women. The overall prevalence of anaemia was 25.8%. The prevalence of anaemia in males was 26.5% and among females, the prevalence was 25.4%. Normocytic anaemia was found in 51.3%, microcytic anaemia was found in 44.9% and macrocytic anaemia was found in 3.8% of the participants. A significant association was observed between anaemia and characteristics such as age, number of comorbidities and diabetes mellitus (*p* < 0.05).

**Conclusion:**

This study revealed that one-fourth of the study population had anaemia. The overall prevalence of anaemia was equal in males and females. The most common type of anaemia was the normocytic normochromic type and there was a significant association between anaemia and characteristics such as age, number of comorbidities and diabetes mellitus.

## Background

Anaemia is a condition of not having sufficient healthy red blood cells or haemoglobin, which is the primary oxygen-carrying molecule, resulting in reduced oxygen delivery to vital organs [1]. As per the World Health Organization (WHO), a condition with haemoglobin levels lower than 13 g/dL for men and 12 g/dL for women indicates anaemia [2]. On the basis of haemoglobin levels, anaemia falls into categories of mild, moderate and severe. Anaemia prevalence rates of 0–20%, 21–40% and over 40% are classified as a public health issue of mild, moderate and severe significance respectively [1]. Anaemia in elderly individuals is a common morbidity, and its incidence grows with age. A national health and nutrition examination survey conducted in the USA from 2013 to 2016 revealed that anaemia affects 65 years old and older people and increases with age for both men and women. The prevalence of anaemia among men aged 65–74 years was 7.4%, whereas for those aged ≥ 85 years it was 39.5%[3]. As per a WHO report, the global prevalence of anaemia among the elderly population is 23.9%[4]. In South-east Asia, the incidence of anaemia among elderly was 24.6% an the highest incidence (45.5%) of anaemia among elderly individuals was observed in Africa [5]. According to a systematic review conducted in India, the combined prevalence of anaemia among the elderly population was 68.3%[6]. The pathogenesis of anaemia is multifactorial and the greater number of comorbidities in elderly patients often makes it difficult to determine the exact aetiology [7]. A decrease in red blood cell production, increased red blood cell destruction and hemorrhage lead to a reduced hemoglobin concentration or red blood cell count and further cause anaemia [8]. The most common symptom of anaemia is fatigue and other prominent signs are pale skin, cold extremities, fast or irregular heartbeat, dyspnoea, chest pain, headaches, dizziness, syncope and hypotension [9]. Anaemia in elderly individuals is often missed for diagnosis as anaemic symptoms such as weakness and exhaustion are construed to be related to natural ageing process [10]. As anaemia is a clinical sign and not a disease it is important to identify and manage the underlying causes. Management strategies are based on the clinical presentation of anaemia. The severity of anaemia is influenced not only by its cause and extent of haemoglobin reduction but also by the presence of other health conditions, the patient’s level of frailty and how quickly anaemia has developed. The factors that contribute to anaemia in older adults include nutritional deficiencies, chronic illnesses, malignancies, and conditions such as leukemia, multiple myeloma and myelodysplastic syndromes. Socioeconomic status and comorbidities also strongly influence the development and severity of anaemia in elderly individuals [11]. Anaemia is a modifiable public health problem, hence early detection and management can prevent adverse outcomes like falls, cognitive decline, and functional dependence. Our study aimed to estimate hemoglobin levels, to classify anaemia on the basis of morphological features and evaluate the factors associated with anaemia among the elderly population above 60 years of age residing in rural areas of Mangalore.

## Methods

### Study design

The present study was conducted in accordance with the Declaration of Helsinki. This community-based cross-sectional study was conducted in rural Mangalore in Dakshina Kannada district, Karnataka. The area is served by the primary health centre (PHC) Kateel, which provides healthcare services to thirteen surrounding villages with a total population of 26,492 and the population of elderly individuals in all thirteen villages is 2172.

## Study population

The study included elderly people over 60 years of age with a study group size of 306 participants. The sample size (n) was calculated considering values [z = 1.96, σ = 1.7, d = 0.2] from a study performed by Pathania et al. in Delhi [12]. The number of study participants required from each of the thirteen villages of Kateel was via non-random (convenient) sampling technique and the required number of elderly individuals from each village was selected. Individuals aged 60 years and above who were permanent residents of the village were included in the study, while those who did not provide consent were excluded. Assistance from village health workers was utilized to facilitate participant contact and engagement.

## Data collection tools

After approval from the Institutional Ethics Committee (IEC), data collection commenced and data were collected by a semi-structured, validated questionnaire. Home visits were performed to obtain the necessary information from these participants. The study purpose and procedure were explained to each participant in detail in their local language and informed consent was obtained. All the information regarding sociodemographic details, dietary details, personal history, functional assessment, morbidity details and investigation details was obtained. The socioeconomic status of the study participants was categorized according to modified BG Prasad’s 2024 classification [13]. A dietary survey of the participants was done by 24-hour recall method and their calorie intake, protein intake and consumption of iron-rich foods were assessed comparing against the Recommended Dietary Allowances (RDA) as per Indian Council of Medical Research (ICMR)-Dietary guidelines for Indians 2024 [14]. The durations alcohol use, smokeless tobacco use and tobacco smoking were recorded. The Functional dependency of the study participants was measured using Katz activities of daily living scale [15]. A history of present conditions such as diabetes, hypertension, thyroid disease, ischemic heart disease and past history of tuberculosis were recorded. Details of the investigation were obtained by assessing the blood samples. For the collection of blood samples, a tourniquet was tied above the cubital fossa and 2 ml of blood was drawn using standard venipuncture technique. The sample was collected in Ethylenediaminetetraacetic acid (EDTA) vial (2 mg/ml) and mixed gently to avoid clotting of blood. The blood samples were collected by a medical doctor trained in phlebotomy, with strict adherence to universal precautions throughout the procedure. The collected samples for each day of data collection were stored in vaccine carrier (temp + 2 to + 8°c) and deposited at Durga Sanjeevani Manipal Hospital, Kateel. Further processing of the samples was carried out at Central Laboratory, Kasturba medical college hospital, Attavar, Mangalore. The time taken from collection of blood sample to processing was approximately 2–3 h. Hemoglobin estimation was performed using Sysmex automated haemoglobin analyser (XN-9000). Anaemia was classified based on morphological features into normocytic normochromic (MCV 80–100 fl.), macrocytic (MCV >100 fl.) and microcytic (MCV < 80 fl.). Factors associated with anaemia were assessed.

### Data analysis

The Data were compiled in an MS Excel and analysed using SPSS version 29.0. Descriptive statistics like median (IQR), proportions and standard deviation were used to express results. The confidence interval for the median is calculated by Bootstrap method. To identify the factors associated with anaemia, logistic regression was used. Variables with p value < 0.2 in the univariate analysis were included in the multivariate analysis.

## Results

The study included 306 elderly people residing in rural areas of Mangalore. The mean age of the study participants was 67.2 ± 7.10 years.

Table 1 shows the baseline characteristics of the study participants. The majority, 224 (73.2%), of the participants were in the 60–70 years age group and about 189 (61.8%) participants were females. A larger proportion of the participants, 240 (78.4%), followed Hindu religion. Most of the participants, 202 (66%), were from nuclear families and 114 (37.1%) participants belonged to the upper middle class. About 283 (92.5%) participants followed mixed diet and 23 (7.5%) participants were vegetarians. Functional status was assessed and majority of the participants 298 (97.4%) were totally independent. Among the participants last occupation prior to retirement, 66.7% (*n* = 204) were engaged in unskilled work, 23.9% (*n* = 73) were engaged in clerical/shop owner/farmer occupations, 6.3% (*n* = 20) were in semi-skilled and skilled occupations while 2.9% (*n* = 9) were semi-professional and professionals. Most of the participants 63.0% (*n* = 193) were married, 32.4% (*n* = 99) were divorced and widowed and 4.6% (*n* = 14) were unmarried. Among the participants, 27.8% (*n* = 85) were illiterates and 26.1% (*n* = 80) had completed high school/intermediate education.

**Table 1:** Baseline characteristics of the study participants (*N* = 306)

Characteristics	*n* (%)
Age group (in years)	
60–70	224 (73.2)
71–80	67 (21.9)
>80	15 (4.9)
Gender	
Male	117 (38.2)
Female	189 (61.8)
Religion	
Hindu	240 (78.4)
Muslim	3 (1.0)
Christian	62 (20.3)
Others	1 (0.3)
Type of family	
Joint	31 (10.1)
Nuclear	202 (66.0)
Three- Generation	73 (23.9)
Socio-economic status*	
Upper class	30 (9.8)
Upper middle class	114 (37.3)
Middle class	85 (27.8)
Lower Middle class	47 (15.4)
Lower class	30 (9.8)
Diet	
Mixed	283 (92.5)
Vegetarian	23 (7.5)
Activities of Daily Living **	
Totally Independent	298 (97.4)
Moderate dependence	5 (1.6)
Severe dependence	3 (1.0)

*Socioeconomic status as per Modified BG Prasad’s classification 2024 [13]

**Katz Index of Independence in Activities of Daily Living (ADL) [15]

**Fig. 1:**
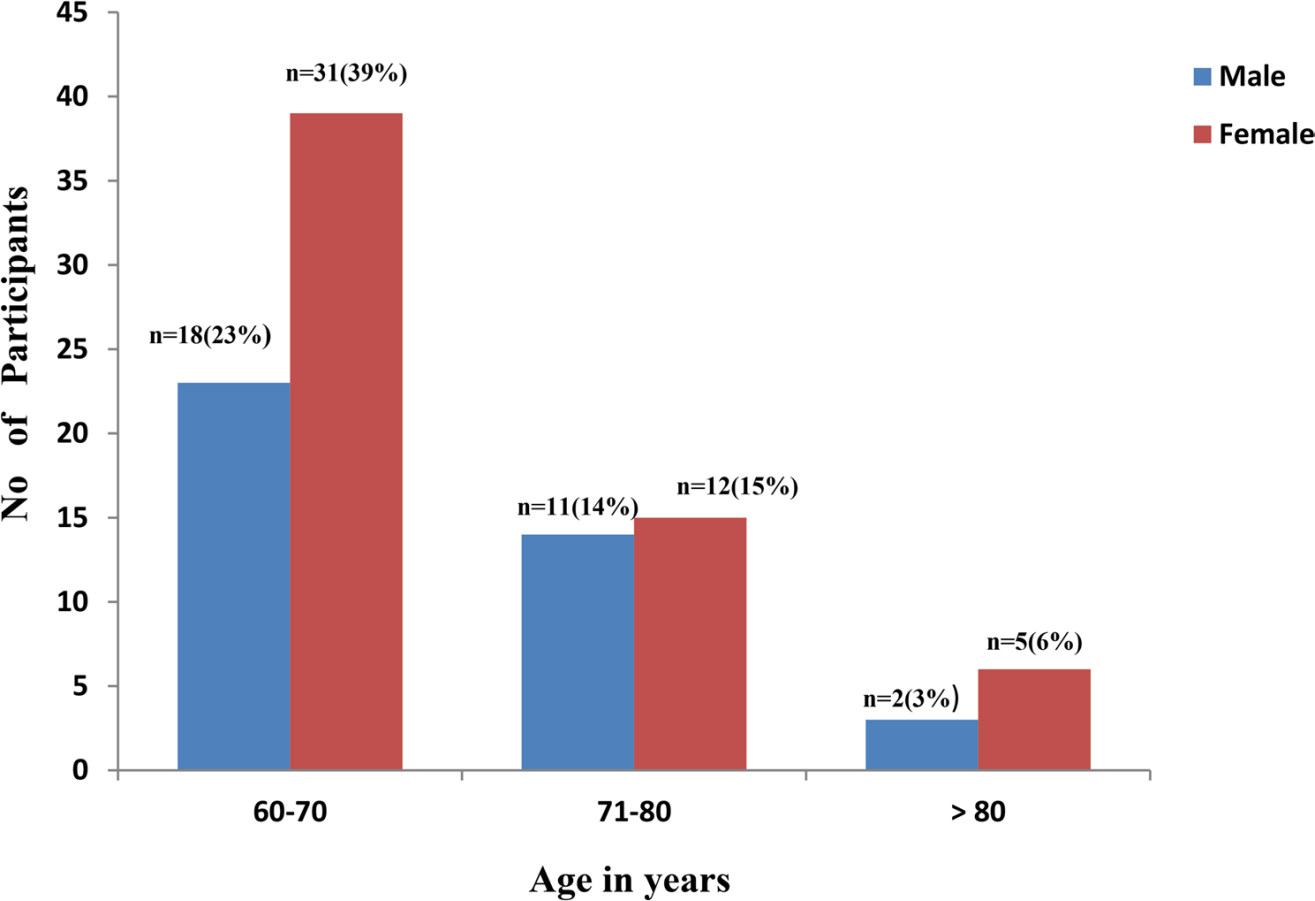
Distribution of Anaemia based on age and gender (*N* = 79)

Figure 1 depicts the distribution of anaemia cases among males and females across three age groups. In the of 60–70 years age group, 18 (23%) males and 32 (39%) females were anaemic, in the 71–80 years age group, 11 (14%) males and 12 (15%) females were anaemic and 2 (3%) males, 5 (6%) females were anaemic in the age group of above 80 years. Among the total participants, 25.8% (*n* = 79) were found to be anaemic. The overall median (IQR) haemoglobin was 13 (12.9–13.2), in men it was found to be 14.0 (13.8–14.1) g/dL and 12.8 (12.7–12.8) g/dL in women. The proportion of anaemia was 26.5% (*n* = 31) among male and 25.4% (*n* = 48) among female participants. The overall prevalence of anaemia among study participants was 25.8% with 95% CI (20.91–30.72). Among the participants, 70.9% (*n* = 56) had mild anaemia, 26.6% (*n* = 21) had moderate anaemia and 2.5% (*n* = 2) had severe anaemia.

**Fig. 2:**
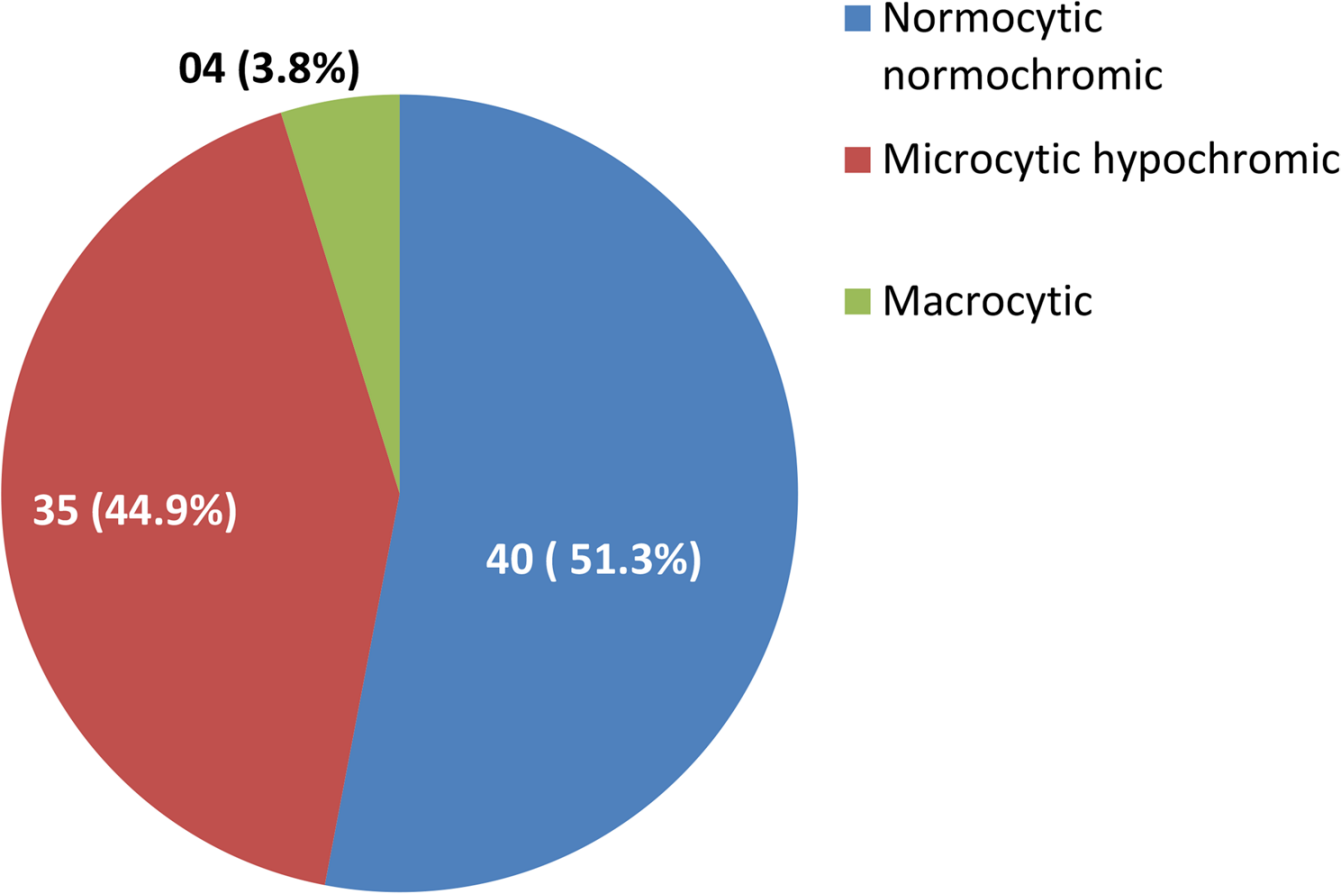
Distribution of anaemia based on the morphological features (*N* = 79

Figure 2 shows the distribution of anaemia based on the morphological features. Among the participants with anaemia, majority, 40 (51.3%), of the participants were found to be having normocytic normochromic anaemia, followed by microcytic anaemia seen in 35 (44.9%) participants and only 4 (3.8%) participants had macrocytic anaemia.

Table 2 shows the association between baseline characteristics and anaemia. Older age was a significant predictor of anaemia, with individuals aged 71–80 years (95% CI: 1.03–3.34, *p* = 0.040) and those above 80 years (95% CI: 1.08–9.04, *p* = 0.036) have higher odds of anaemia than those aged 60–70 years. There was no significant association between anaemia and other baseline characteristics such as type of family, socio-economic status and functional dependency.

**Table 2:** Association between baseline characteristics and anaemia (*N* = 306)

Anaemia						
	Present *N* = 79 *n* (%)	Absent *N* = 227 *n* (%)	*P* value	Total	Unadjusted OR (95%CI)	*P*- value
Age category (in years)						
60–70	49 (21.9)	175 (78.1)	0.021	224	Reference	
71–80	23 (34.3)	44 (65.7)		67	1.867 (1.029 − 3.338)	0.040
> 80	7 (46.7)	8 (53.3)		15	3.125 (1.080 − 9.044)	0.036
Type of family						
Three- Generation	11 (35.5)	20 (64.5)	0.351	31	Reference	
Nuclear	52 (25.7)	150 (74.3)		202	1.959 (0.780 − 4.923)	0.152
Joint	16 (21.9)	57 (78.1)		73	1.235 (0.653 − 2.337)	0.517
Socio-economic status						
Upper class	6 (20.0)	24 (80.0)	0.869	30	Reference	
Upper middle class	29 (25.4)	85 (74.6)		114	1.365 (0.508 − 3.669)	0.538
Middle class	21 (24.7)	64 (75.3)		85	1.313 (0.473 3.645)	0.602
Lower Middle class	14 (29.8)	33 (70.2)		47	1.697 (0.570–5.054)	0.342
Lower class	9 (30.0)	21 (70.0)		30	1.714 (0.523 − 5.621)	0.374
Functional Dependency						
Totally Independent	75 (25.2)	223 (74.8)	0.201	298	Reference	
Mild, Moderate dependence	2 (40.0)	3 (60.0)		5	1.982(0.325–12.091)	0.458
Severe dependence	2 (66.7)	1 (33.3)		3	5.947 (0.532 - 66.520)	0.148

*p value < 0.05 is considered statistically significant

Table 3 shows the association of dietary characteristics with anaemia. The greater proportion of anaemia was observed among the participants with inadequate calorie intake (*n* = 64, 27.1%), participants with inadequate protein intake (*n* = 54, 29.5%) and participants with intake of iron rich foods less than three times a week(*n* = 45, 29.4%). No significant associations were found between anaemia and dietary characteristics such as the type of diet, calorie intake, protein intake and intake of iron rich foods.

**Table 3:** Association between dietary characteristics and anaemia (*N* = 306)

	Anaemia					
	Present *N* = 79 *n* (%)	Absent *N* = 227 *n* (%)	*P* value	Total	Unadjusted OR (95%CI)	*P* - value
Diet						
Mixed	76 (26.9)	207(73.1)	0.146	283	Reference	
Vegetarian	3 (13.0)	20 (87.0)		23	0.409 (0.118–1.414)	0.158
Calorie intake						
Adequate	15 (21.4)	55 (78.6)	0.339	70	Reference	
Inadequate	64 (27.1)	172(72.9)		236	1.364 (0.720–2.585)	0.341
Protein intake						
Adequate	25 (20.3)	98 (79.7)	0.072	123	Reference	
Inadequate	54 (29.5)	129 (70.5)		183	1.641 (0.954–2.822)	0.073
Intake of rich iron foods **≥** 3 times a week						
Yes	34 (22.2)	119(77.8)	0.151	153	Reference	
No	45 (29.4)	108(70.6)		153	0.929 (0.556–1.551)	0.777

*p value < 0.05 is considered statistically significant

Table 4 shows the association of morbidity characteristics and anaemia. Participants with higher number of comorbidities had increased odds of anaemia, with two comorbidities (95% CI: 1.12–3.98, *p* = 0.021) and those with three comorbidities (95% CI: 1.36–14.21, *p* = 0.013), compared to individuals without comorbidities. In addition, diabetes mellitus was identified as a significant risk factor for anaemia (OR: 1.96, 95% CI: 1.16–3.30, *p* = 0.012). No significant associations were observed for other comorbidities such as hypertension, ischemic heart disease or thyroid disorders.

**Table 4:** Association between comorbidities and anaemia (*N* = 306)

	Anaemia					
	Present *N* = 79 *n* (%)	Absent *N* = 227 *n* (%)	*P* value	Total	Unadjusted OR (95%CI)	*P*- Value
Comorbidities						
Present	53 (29.1)	129 (70.9)	0.110	182	1.549 (0.904–2.652)	0.111
Absent	26 (21.0)	98 (79.0)		124	Reference	
No. of comorbidities present						
None	26 (21.0)	98 (79.0)	0.005	124	Reference	
1	18 (19.8)	73 (80.2)		91	0.929 (0.474–1.822)	0.831
2	28 (35.9)	50 (64.1)		78	2.111 (1.121–3.976)	0.021
3	7 (53.8)	6 (46.2)		13	4.397 (1.361–14.211)	0.013
Diabetes Mellitus						
Yes	38 (34.2)	73 (65.8)	0.011	111	1.955 (1.160–3.295)	0.012
No	41 (21.0)	154 (79.0)		195	Reference	
Hypertension						
Yes	44 (31.0)	98 (69.0)	0.055	142	1.655 (0.988–2.772)	0.056
No	35 (21.3)	129 (78.7)		164	Reference	
Ischemic heart disease (IHD)						
Yes	6 (37.5)	10 (62.5)	0.273	16	1.784 (0.626–5.078)	0.278
No	73 (25.2)	217 (74.8)		290	Reference	
Thyroid disorder						
Yes	7 (46.7)	8 (53.3)	0.058	15	2.661 (0.933–7.596)	0.067
No	72 (24.7)	219 (75.3)		291	Reference	

*p value < 0.05 is considered statistically significant

Table 5 shows the multivariate analysis of various predictors of anaemia. In Model 1,after adjusting for variables, having three comorbidities was a significant predictor of anaemia, with 4.85 higher odds than those without comorbidities (AOR: 4.85, 95% CI: 1.44–16.35, *p* = 0.011). In Model 2, anaemianot significantly associated with variables such as age, type of family, functional dependency, diet, protein intake, diabetes and hypertension.

**Table 5:** Multivariable analysis of predictors of anaemia in the elderly population

	Model 1 with number of comorbidities		Model 2 with HTN, DM and Thyroid disorders	
Characteristic	Adjusted OR (95% CI)	*P*- Value	Adjusted OR (95% CI)	*P*- Value
Age category (in years)				
60–70	Reference		Reference	
71–80	2.0394 (0.0549- 1.370)	0.034	2.0243 (0.0482–1.362)	0.035
> 80	2.9999 (−0.1740- 2.371)	0.091	3.1243 (−0.1230–2.401)	0.077
Gender				
Male	Reference		Reference	
Female	1.0993 (− 0.4987–0.688)	0.754	1.1407 (−0.4596–0.723)	0.663
Type of family				
Three- Generation	Reference		Reference	
Nuclear	2.0672 (−0.2461- 0.688)	0.143	1.9061 (−0.3264 −1.617)	0.193
Joint	1.6320 (−0.2913- 1.271)	0.219	1.6145 (−0.2922- 1.250)	0.223
Socio-economic status				
Upper class	Reference		Reference	
Upper middle class	1.8635 (−0.5248–1.770)	0.288	2.0344 (−0.4422- 1.863)	0.227
Middle class	2.3929 (−0.3874-2.132)	0.175	2.6357 (−0.3000- 2.238)	0.134
Lower Middle class	2.2503 (− 0.5127–2.135)	0.230	2.5679 (−0.3798–2.266)	0.162
Lower class	1.0709 (−1.3479-1.485)	0.924	1.1913 (−1.2465- 1.597)	0.809
Functional Dependency				
Totally Independent	Reference		Reference	
Mild, Moderate dependence	2.5424 (−1.1592- 3.025)	0.382	2.1047 (−1.3260- 2.814)	0.481
Severe dependence	4.7999 (−1.2604- 4.398)	0.277	5.2314 (−1.2375- 4.547)	0.262
Diet				
Mixed	Reference		Reference	
Vegetarian	0.4376 (−2.1283 −0.476)	0.213	0.4458 (−2.0965- 0.481)	0.219
Protein intake				
Adequate	Reference		Reference	
Inadequate	1.3800 (−0.2907–0.935)	0.303	1.3406 (−0.3187- 0.905)	0.348
Diabetes Mellitus				
Absent	-		Reference	
Present	-	-	1.5808 (−0.1582- 1.074)	0.145
Hypertension				
Absent	-		Reference	
Present	-		1.2884 (−0.3358- 0.843)	0.399
Thyroid disorders				
Absent	-	-	Reference	
Present	-		2.5627 (−0.2129- 2.095)	0.110
No. of comorbidities present				
None	Reference			
1	1.0027 (−0.7088–0.714)	0.994	-	
2	1.8529 (−0.0556- 1.289)	0.072	-	
3	5.5703 (0.4771–2.958)	0.007	-	

*As the total number of comorbidities and specific conditions such as hypertension, diabetes, and thyroid disorders are interdependent, a separate model was developed

## Discussion

Anaemia is a common, often underdiagnosed condition in elderly individuals who contributes significantly to morbidity, decreased quality of life, and increased health care burden. Age-related physiological changes and comorbidities increase the vulnerability of elderly individuals to anaemia. The coexistence of conditions such as heart failure and compromised cerebral circulation further reduces the ability of elderly individuals to tolerate anaemia. The symptoms of anaemia in elderly individuals are often overlooked. Our study aimed assess the prevalence of anaemia and to evaluate the factors associated with anaemia among the elderly population above 60 years of age residing in rural areas of Mangalore.

In our study, three fourths of the participants were in the 60–70-year age group. Similar studies done by Krishna Murthy et al. in Tamil Nadu [16] and Shrivastava S et al. in Karnataka [17] revealed that most of the participants were aged 60–70 years. In contrast, in another study done by Pathania et al. in Delhi, [12] the largest group of participants were aged 70–79 years. In our study, three fifths of the participants were females. This may be due to more women being present at home at the time of data collection during the day. Other studies, done by Gupta S et al. in Haryana [10] and Retnakumar et al. in Kochi [18] had similar findings of participation of greater proportion of females. In our study, it was observed that four fifth of the participants were Hindus. Similarly, in a study done by Gupta A et al. in Uttarakhand, [19] the majority of the participants were Hindus. In the present study, three-fifths of the participants were married and were from nuclear families. In another study done by Pathania et al. in Delhi, [12] the majority of the participants also were also from nuclear families. In our study, the functional dependence of the study participants was measured by Katz Activities of Daily Living. A total of 97.4% of the participants were totally independent, while 1.6% of the participants had moderate dependence and only 0.1% participants had severe dependence. This could be because the majority of the study participants were 60–70 years old. In contrast, studies done by Agarwalla et al. in rural Assam, [20] Wang et al. in China, [21] Onem Y et al. in Istanbul, Turkey [22] and Bang S et al. in Korea, [23] reported equal distributions of study participants in both functionally independent and functionally dependent categories. In our study the overall median (IQR) haemoglobin levels among men and women were 14.0 (12.9–15.1) and 12.8 (11.9–13.5) (gm/dl) respectively. Our findings are in line with various studies done by Karoopongse E et al. in Thailand [Mean Hb in males 13.9(1.36) gm/dl and mean Hb in women 12.6 (1.12) gm/dl] [24]. In contrast, in studies done by Sharma D et al. in Chandigarh [Mean Hb 8.8(2.3)gm/dl], [25] Pathania et al. in Delhi [overall mean Hb 11.6(1.7) gm/dl, mean Hb among men 12.1(1.7) gm/dl, mean Hb in women 10.9(1.6)gm/dl], [12] Krishnamurthy S et al. in Coimbatore[Mean Hb 7.1 gm/dl] [16] and by Gupta A et al. in Uttarakhand [Mean Hb in men 10.9(1.0) gm/dl, among women [9.9(1.5)gm/dl], [19] low mean Hb levels were observed. In our study, the highest (46.7%) prevalence of anaemia was observed in age above 80 years age, possibly because, as the age advances, the production of haematopoietin decreases leading to anaemia [26]. Our study findings are in line with studies done by Pathania A et al. in Delhi, [12] Gupta A et al. in Uttarakhand, [19] Debnath A et al. in West Bengal [27] and Yildirim T et al. in Ankara, Turkey [28]. In this study, the overall anaemia prevalence was 25.8% and an equal prevalence of anaemia was observed among males and females. The prevalence of anaemia in the present study was lower than that reported in various studies done across India such as studies done by Debnath et al. in West Bengal (65%), [27] Retnakumar et al. in Kochi(60.6%), [18] Pathania et al. in Delhi (68.7%), [12] and Agarwalla et al. in Assam (45.5%) [20]. This may be due to the fact that majority (four-fifths) of the participants followed mixed diet and the nutrition is the significant predictor of hemoglobin status. Non-vegetarian diets are rich in vitamin B12, which is crucial for red blood cell formation, has heme iron and is absorbed more efficiently than non-heme iron found in plant sources. In the present study, two-fifths belongs to upper socioeconomic (SES) class. This may be due to that individuals with higher SES are generally less likely to be anaemic, whereas those with lower SES are more likely to have anaemia. SES plays a crucial role in influencing the prevalence, severity, and management of anaemi across populations [29]. Similarly in a study done by Gupta A et al. in Uttarakhand [19] only 17% of participants belonging to upper socioeconomic class were found to be anaemic and in a similar study done by Paul S et al. in Vellore [30] only 34.3% of participants from middle and higher socioeconomic were anaemic. In our study, one-fourth of the participants had education up to high school/intermediate. Education influences anaemia through health-related behaviours, as educated individuals tend to have better awareness of nutritional requirements, healthcare utilization, and preventive practices. Our study findings are consistent with studies done by Gupta S et al. in Haryana [10] where 30% participants had anaemia and in a study done by Lohmror A et al. in Jaipur, [31] 11.8% participants had anaemia.

In this study, the prevalence of normocytic normochromic anaemia was highest, followed by microcytic and macrocytic anaemia. Our findings are in line with studies done by Krishnamurthy et al. in Coimbatore (49%), [16] Bhasin A et al. in Bangalore (62%), [32] Sharma et al. in Chandigarh (53.3%), [25] Srivastava S et al. in Bijapur (78%), [17] Mann S et al. in Dehradun (50%), [33] Khatib W et al. in Maharashtra (43%), [34] Gangadharan V et al. in Andhra Pradesh (73%), [35] Greeshma C.G et al. in Chennai (40%), [36] Prakash K et al. in Bangalore (52%) [37] and Bach et al. in Europe (78%) [38]. On the basis of various studies conducted, the most common cause of normocytic normochromic anaemia was anaemia of chronic disease followed by hemolytic anaemia.

In the present study, the prevalence of mild anaemia was the highest followed by moderate and severe anaemia. Our study findings are consistent with various studies done by Debnath et al. in West Bengal (41.6%), [27] Gupta S et al. in Haryana (26.7%), [10] Deore B et al. in Nashik Maharashtra (76.8%), [39] and Gangadharan V et al. in Andhra Pradesh (40%), [35] The most common reason for mild anaemia is that it often presents without noticeable symptoms, and when symptoms such as fatigue or weakness do occur, they are typically mild and therefore frequently overlooked. In contrast, a study done by Mann S et al. in Dehradun (46.67%) [33] reported moderate anaemia and in a study done by Prakash K et al. in Bangalore (42%) [37] reported severe anaemia.

In the present study, there was an association between anaemia and the number of comorbidities. A greater proportion of anaemia was observed in patients with maximum number of comorbidities. Our findings are consistent with studies done by Gandhi S et al. in Camden, USA, [40] Domenica Cappellini M et al. in Italy [8]. 

Early detection and timely treatment of anaemia among elderly individuals further prevents complications, decreases morbidity and improves quality of life despite having various coexisting comorbidities.

## Limitations of the study

The study follows a cross-sectional study design; it may not establish causality of anaemia. As the study was conducted in a relatively small population using non probability sampling technique, which limits the generalizability of the findings. The 24-hour recall method for dietary assessment is a limitation, as it captures only a single day’s intake and is therefore insufficient to represent an individual’s usual long-term dietary habits due to day-to-day variations.

## Conclusion

Our study revealed that one-fourth of the study population had anaemia. The overall prevalence of anaemia was equal in males and females. The most common type of anaemia was normocytic normochromic anaemia followed by microcytic anaemia. There was a significant association between anaemia and characteristics such as age, number of comorbidities and diabetes mellitus. The burden of anaemia can be effectively reduced by conducting community-level health education sessions that emphasize lifestyle changes, routine screening of elderly individuals, the need to adhere to treatment for existing conditions, and nutritional awareness, especially about the use of inexpensive, locally accessible, and nutrient-dense foods.

## Data Availability

The datasets used and/or analysed during the current study available from the corresponding author on reasonable request.

## Abbreviations

ADL: Activities of daily living
EDTA: Ethylenediaminetetraacetic acid
IEC: Institutional ethics committee
SES: Socio economic status
WHO: World health organisation
